# Comparative Analytics and Pharmacodynamics of the Complex Protein-Free Botulinum Toxin Type A Formulations DaxibotulinumtoxinA, IncobotulinumtoxinA and RelabotulinumtoxinA

**DOI:** 10.3390/toxins18030142

**Published:** 2026-03-14

**Authors:** Stefanie Honndorf, Katja Kühbach, Karl-Heinz Eisele, Alina Shokurova, Philipp Buch, Claudia Jatzke, Harold Victor Taylor, Klaus Fink

**Affiliations:** Merz Therapeutics GmbH, Neurotoxin & Biotechnology Development, Eckenheimer Landstr. 100, 60318 Frankfurt, Germany

**Keywords:** botulinum toxin type A, BoNT/A, IncobotulinumtoxinA, DaxibotulinumtoxinA, RelabotulinumtoxinA, mouse digit abduction score

## Abstract

Botulinum neurotoxin type A (BoNT/A) is intramuscularly injected for the treatment of, e.g., spasticity, cervical dystonia or facial lines. Several BoNT/A products with or without complexing proteins, with non-interchangeable dose units and various duration of effect claims, are approved but hard to compare. The goal of this study was to compare the complexing protein-free approved BoNT/A products IncobotulinumtoxinA (INCO), DaxibotulinumtoxinA (DAXI) and RelabotulinumtoxinA (RELA) in vitro and in vivo. BoNT/A protein content per 100 U was lowest in INCO and highest in DAXI (INCO 0.44, RELA 0.46, DAXI 0.58 ng/100 U). Relative bioactivity of INCO, DAXI and RELA was comparable (116, 104 and 117 U/100 labeled units). INCO and DAXI caused a maximum mouse digit abduction score (DAS) 2–3 days after IM injection of 20 or 40 U/kg. The DAS after 20 U/kg INCO was higher and showed a 10 days longer paralysis than DAXI at equivalent dosing. The in vivo spread of DAXI in the mouse gastrocnemius muscle was indistinguishable from that after INCO, and the spread of RELA ex vivo in porcine muscle was larger than INCO but equal to 0.9% NaCl. These results show the differences between 150 kDa botulinum type A toxin products beyond the published claims.

## 1. Introduction

In the present study, we analyzed the three BoNT/A products INCO, RELA and DAXI in vitro for their bioactivity and neurotoxin protein content. We also compared the two lyophilized BoNT/A products INCO and DAXI in vivo in a mouse digit abduction score (DAS) after single intramuscular (IM) administration of equivalent doses. The level and duration of paralysis were determined over a 42-day period in order to compare the performance of the products. Finally, the spread of DAXI vs. INCO was assessed by in vivo imaging and that of RELA vs. INCO by ex vivo volumetry.

Botulinum neurotoxin A (BoNT/A) blocks the release of acetylcholine from nerve terminals at the neuromuscular junction, resulting in reversible, flaccid paralysis. This activity is used for the treatment of therapeutic or aesthetic clinical indications. These therapeutic conditions comprise muscle hyperactivity, including post-stroke spasticity, cerebral palsy and cervical dystonia requiring regular, quarterly to semi-annual injections. Especially in pediatric spasticity patients, high dosages are required to avoid pain and secondary disabling malformations or at least confer some usability to the spastic limb [1,2,3,4]. For those patients BoNT/A formulations must provide a high safety level and long-term efficacy.

Currently, several 150 kDa BoNT/A products free of neurotoxin-associated complex proteins are commercially available. These include INCO (IncobotulinumtoxinA, Xeomin^®^, Merz Pharmaceuticals GmbH, Frankfurt am Main, Germany), DAXI (DaxibotulinumtoxinA, DAXXIFY^®^, Revance Therapeutics Inc., Newark, NJ, USA) and RELA (RelabotulinumtoxinA, Relfydess^®^, Galderma, Uppsala, Sweden). The prefixes Inco, Daxi and Rela are specific identifiers given by the United States Adopted Names (USAN) Council. Although all three formulations contain similar drug substances, the manufacturing steps and the excipients may contribute to differences in therapeutic efficacy and duration of action. Each manufacturer initially bases their dose unit upon a proprietary mouse lethality assay even if most then change to an in vitro cell-based potency assay. These proprietary assays result in unique dose units for each individual product making them non-interchangeable [5,6]. Unit determination, as well as the different excipients and manufacturing processes consequently influence the therapeutic dose and treatment interval needed to achieve the desired benefits in patients [6]. The selection of the BoNT/A formulation by the physician is crucial because it may confer longer efficacy for the patients without increased side effects.

Since its first registration in Germany in 2005, INCO has been approved for therapeutic and aesthetic indications. These include cervical dystonia, spasticity and blepharospasm as well as glabellar frown lines and crow’s feet [7,8,9]. INCO is a lyophilized product, which contains the 150 kDa neurotoxin free of complexing proteins, 1 mg human serum albumin (HSA) and 4.7 mg sucrose per 100 U vial [9,10]. Although the dose units are not interchangeable between BoNT/A products, the potency of INCO has been compared with earlier registered BoNT/A products OnabotulinumtoxinA (ONA) or AbobotulinumtoxinA (ABO) in therapeutic and aesthetic clinical trials [11,12,13,14,15,16,17]. The authors concluded that the unit conversion factor for ONA and INCO is unity. In contrast, the conversion factor between ONA (and thus INCO) and ABO was suggested to be 1:3 to 1:4 [18,19].

The second BoNT/A formulation investigated here, DAXI, is also a lyophilizate and was approved by the FDA in 2022 for the treatment of upper facial lines and in 2023 for the treatment of cervical dystonia. The excipients in DAXI include a synthetic cationic peptide named RTP004, the surfactant polysorbate-20, histidine, histidine-HCl-monohydrate, and the sugar trehalose dihydrate [20,21]. RTP004 is a 35-amino acid synthetic peptide. It consists of 15 lysine residues flanked on either side by the protein transduction domain (PTD) [20,22] of the human immunodeficiency virus (HIV-1) Tat protein (RKKRRQRRRG-K15-GRKKRRQRRR) and is positively charged at pH 7.4. The PTD sequence allows the HIV protein to penetrate the plasma membrane of target host cells [23,24,25]. The cationic peptide is supposed to interact non-covalently with the negatively charged BoNT/A surface, stabilizing the protein at room temperature and thus preventing protein aggregation and adsorption to surfaces. RTP004’s positive charge was postulated to facilitate DAXI’s binding to negatively charged presynaptic nerve terminals, as well as neuronal surfaces, while facilitating the localization of the product for internalization and reducing its diffusion from injection sites, leading to increased neurotoxin uptake into the neuron and thus prolonged efficacy [20,22]. If the hypothesis would translate into real-life therapy, it should confer higher efficacy per unit compared to other BoNT/A preparations. We tested the efficacy claim in vitro in a cell-based potency assay the confinement claim by in vivo imaging after intramuscular injection in mouse and the potency and duration claims by mouse digit abduction scoring (DAS).

In clinical practice, a Phase 2 trial for the treatment of moderate and severe glabellar lines DAXI (40 U) was compared to the approved dose of ONA (20 U). The data revealed that double the BoNT/A dose in DAXI had a stronger effect and 1.23 times longer duration in patients (time to loss of a ≥1-point improvement on the Investigator Global Assessment–Frown Wrinkle Severity score) compared to 20 U ONA [26,27]. Therapeutic use in a clinical Phase 3 study in cervical dystonia showed 20.3 weeks median duration of effect when treating patients with 250 U DAXI [28], which is longer than the median effect duration of 12–14 weeks reported for ONA (median total dose 236 U), ABO (start dose 500 U, afterwards titration in range of 250–1000 U) or INCO (240 U) [29,30,31]. The median effect duration was determined by the time to loss of 80% peak treatment benefit. However, the DAXI dose was higher compared to INCO and ONA, which might be the cause for the higher median duration of effect in the trial.

The third formulation RELA investigated here is a ready-to-use liquid formulation. It was approved in Europe and Australia in 2024 for the treatment of moderate-to-severe glabellar lines and lateral canthal lines in adults. The excipients in RELA include the surfactant polysorbate 80, disodium hydrogen phosphate dihydrate, sodium, dihydrogen phosphate dihydrate, potassium chloride, sodium chloride and L-tryptophan [32,33,34]. The BoNT/A in RELA is purified without precipitation steps but, similar to INCO, by column chromatography and filtration to keep denaturing of the neurotoxin low [32,33]. In a comparison of RELA and ONA based not on bioactivity units but on the recommended doses for glabellar frown line treatment, i.e., 50 U RELA and 20 U ONA, RELA had, accordingly, a higher amount of neurotoxin protein (0.27 ng) than ONA (0.18 ng), a higher catalytic activity per glabellar line dose (ratio 53 vs. 29) and a higher bioactivity with cleaved SNAP-25 11% vs. 6% after 8.5 h incubation indicating a stronger effect of the higher toxin dose in RELA [35]. While the result is not surprising, it only indicates more inactive neurotoxin in ONA than in RELA.

## 2. Results

### 2.1. Concentration and Biologic Activity of BoNT/A

For the head-to-head analysis of the three BoNT/A formulations, the BoNT/A content was first determined using an enzyme-linked immune-sorbent assay (ELISA) specific for BoNT/A detection and validated for INCO. We analyzed three batches of INCO or RELA and one DAXI batch. The nominal units for INCO, DAXI and RELA are 100 U, 100 U and 150 U per vial, respectively. For reasons of comparability, the RELA results were calculated to 100 labeled units/mL and are further not distinguished from the 100 U/vial used for INCO and DAXI.

The BoNT/A protein content in INCO, DAXI and RELA was 0.44, 0.58, and 0.46 ng/100 U, respectively (Table 1). Table 1 lists the compositions of all three of these commercial products [9,21,34].

Since the BoNT/A ELISA was validated by the authors specifically for INCO, its fitness for purpose was confirmed for DAXI and RELA prior to use. Both formulations contain excipients such as PS80 or the synthetic peptide RTP004 with potentially unknown effects. Samples of either formulation were spiked with BoNT/A and the results tested for equivalence to ensure validity of results. At least 11 valid measurements were obtained for each formulation. For each of the two test products, the difference in measurement results of test matrix vs. validated matrix was calculated. At a significance level of 0.05, the measured BoNT/A concentrations of the samples composed for the DAXI or RELA samples were equivalent within the limits of +/−7% of the concentrations determined in the samples with validated sample matrix, demonstrating that the excipients of DAXI and RELA have no effect on the ELISA detection method.

**Figure 1:**
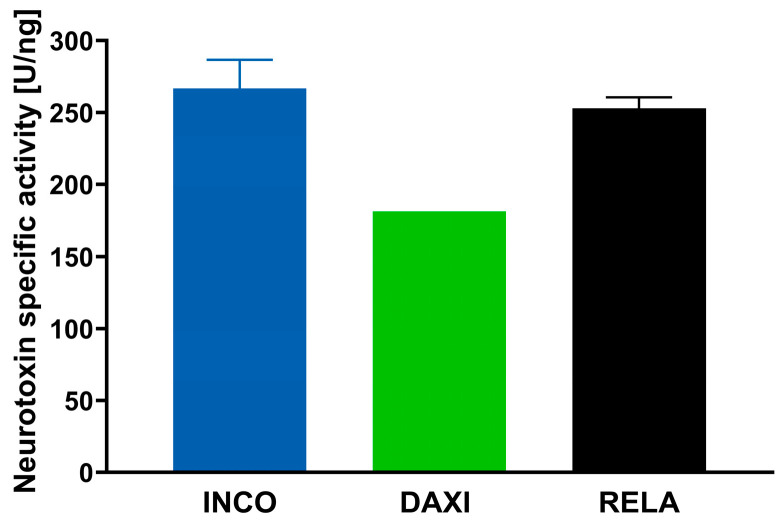
Specific neurotoxin activity of INCO, DAXI and RELA in U/ng after detection of protein content and relative bioactivity per 100 U in an ELISA and cell-based assay. Mean + SD, *n* = 1–4.

As ELISAs do not distinguish active from inactive proteins, the bioactivity of BoNT/A was also determined in each formulation. The mean biological activity of the product batches was 116.0 U/vial for INCO, 104.2 U/vial for DAXI and 116.9 U/vial for RELA corresponding to 100 labeled units. The mean specific potency was 266.7 U/ng for INCO, 181.1 U/ng for DAXI and 252.9 U/ng for RELA (Figure 1).

Bioactivity was determined by a cell culture assay based on human-induced pluripotent stem cell (iPSC)-derived neuronal cells. To test these cells for compatibility with the excipients, they were exposed to polysorbate 20 (PS20), polysorbate 80 (PS80), RTP004, D-(+)-trehalose dihydrate, L-tryptophan, L-histidine, sodium dihydrogen phosphate dihydrate and the combinations, thereof simulating the commercial formulations. Subsequently, the cells were checked for viability and membrane integrity. While PS80 and RTP004 are cytotoxic to the neuronal cells of the cell-based assay when applied undiluted, at the 1:250 dilution level used, no excipient or simulated formulation had cytotoxic effects on the neuronal cells.

### 2.2. Digit Abduction Score

**Figure 2:**
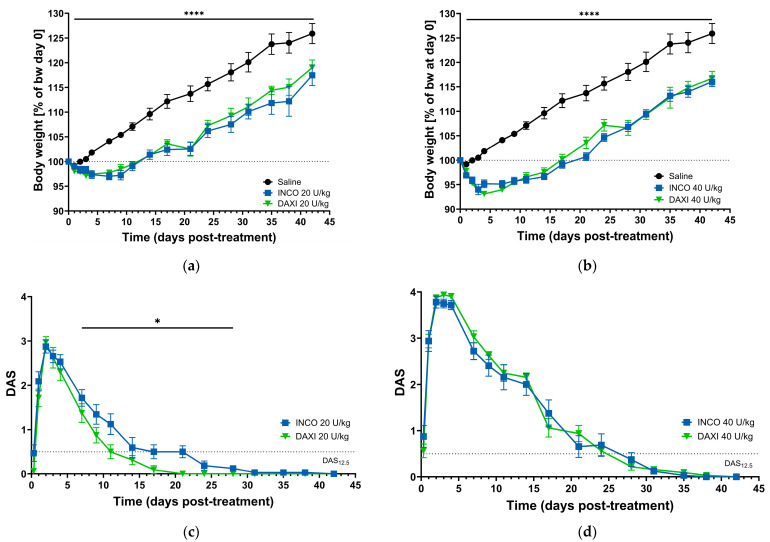
Effect of INCO and DAXI on body weight and digit abduction score. (**a**,**b**) Relative mean body weight after INCO and DAXI injection ((**a**), BoNT/A 20 U/kg; (**b**), 40 U/kg). BoNT/A-treated groups demonstrated a dose-dependent initial decrease in body weight followed by weight gain after day 7 (20 U/kg) or 4 (40 U/kg) (treated vs. placebo **** *p* < 0.0001 mixed-effects model/two-way RM ANOVA). Mean ± SEM, *n* = 7–8. (**c**,**d**) INCO caused a higher DAS response than DAXI from day 4 to day 28 (mixed-effects ANOVA model * *p* < 0.05 for day 7–28). INCO and DAXI did not differ in DAS response at 40 U/kg (*p* > 0.05). The dotted line at DAS = 0.5 represents 12.5% of the max DAS response and is used for duration of action calculation at this point. Mean ± SEM, *n* = 7–8. Initial bodyweight was in the saline group 22.2 ± 1.5 g, INCO 20 U/kg 22.0 ± 0.7 g, INCO 40 U/kg 21.0 ± 1.1 g, DAXI 20 U/kg 20.7 ± 1.4 g, DAXI 40 U/kg 22.3 ± 1.6 g.

The injection of BoNT/A elicited a short-term loss of body weight in the verum study groups for the first week post-injection. This weight loss occurred in a dose-dependent manner, with 20 and 40 U/kg resulting in up to 3.0% and 6.0% mean loss in the INCO group and 3.3% and 7.0% in the DAXI group, respectively (verum versus placebo; * *p* < 0.0001 for 20 and 40 U/kg). The maximum weight loss occurred in the high dose DAXI group with 6.9% on the fourth day after administration. Following this initial loss, the weight in all BoNT/A dose groups increased at similar rates (Figure 2a,b).

The digit abduction score was used to quantify the BoNT/A-elicited flaccid paralysis of neighboring muscles upon the injection of BoNT/A into the *m. gastrocnemius.* The DAS is frequently employed to assess the biological activity and persistence of BoNT/A products [36,37]. Accordingly, it was performed to investigate if the differences in formulation have an impact on the paralytic effect. Following placebo treatment, mice exhibited normal or uninhibited digit abduction (DAS = 0) for the duration of the study. In contrast, the INCO and DAXI treatment groups exhibited inhibition of digital abduction as a function of the applied dosage (Table 2).

Both BoNT/A formulations displayed the maximum DAS response 2–3 days after IM injection of 20 or 40 U/kg. Administration of 20 U/kg INCO led to an increased paralysis in comparison to DAXI over the entire study period except for days 2 and 42, as indicated by an upwards shift in the mean DAS response. The paralytic effect was significantly higher for INCO from day 7–28 (* *p* < 0.05). In contrast, INCO and DAXI groups injected with 40 U/kg did not differ in their DAS response (Figure 2c,d). The higher average digital abduction score in the low dosage INCO group equals a ten day longer persistence of paralysis, i.e., before returning to DAS_12.5_ (DAS = 0.5). This higher digital abduction scoring is the consequence of a stronger paralysis, as opposed to that generated by DAXI in this time frame (Figure 2c). In both high dose groups, the duration of action until return to DAS_12.5_ was 24–27 days after INCO or DAXI administration.

The area under the DAS curves over time (DAS_AUC_) was significantly increased by 51.6% in the 20 U/kg INCO group compared to the DAXI group (* *p* < 0.05, Table 2), whereas at 40 U/kg the DAS_AUC_ did not differ between INCO and DAXI.

### 2.3. Far-Red Fluorescence (FRF) In Vivo Imaging

**Figure 3:**
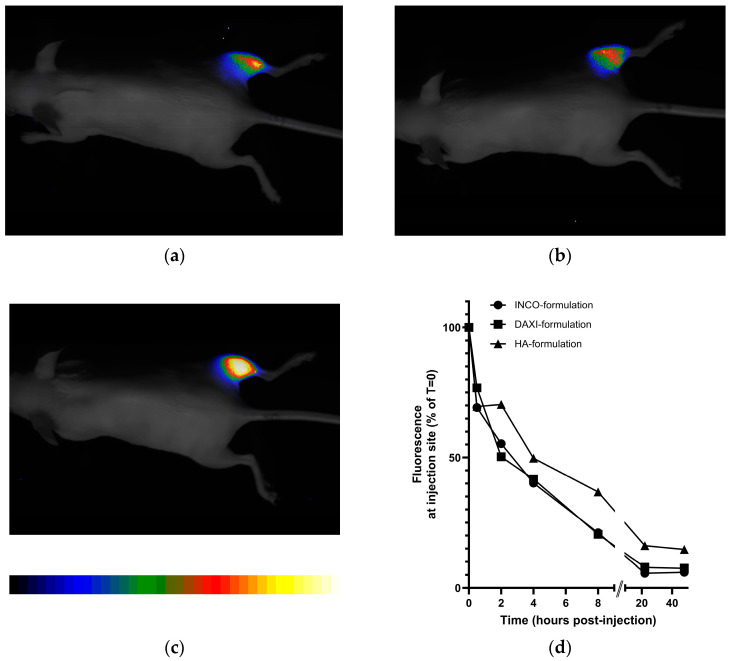
In vivo imaging of far-red fluorescence (FRF)-labeled, inactive fiBoNT/A spreading after injection of 10 µg in 20 µL into right gastrocnemius muscle of mice. Fluorescence intensity is shown in pseudo-colors (blue–green–red–yellow = ascending fluorescence brightness). Overlay of position control picture (gray) and FRF image (pseudo-colors). The exemplary images shown were taken 4 h post-injection of fiBoNT/A in (**a**) INCO, (**b**) DAXI and (**c**) non-commercial HA formulation; (**d**) time course of fluorescence intensity at the injection site after application of 10 µg fiBoNT/A in 20 µL of INCO, DAXI or HA formulation.

Inactive BoNT/A was chemically labeled with an infrared fluorescent dye (fiBoNT/A). After injection, the fluorescent dye is clearly located in the injection area, i.e., the gastrocnemius region of the right hind limb (Figure 3a–c). The quantitative post-injection time course of the remaining fluorescence at the injection site is shown in Figure 3d. The fluorescence intensity at the injection site decreases over a time period of approx. 24 h. For the INCO and DAXI formulation, virtually no difference could be observed in spreading kinetics with a half-life of approx. 3.5 h. Interestingly, if formulated with 5 mg/mL hyaluronic acid, the fiBoNT/A persisted considerably longer at the injection site with a half-life of 6.4 h.

### 2.4. BoNT/A Spread in Muscle Tissue

**Figure 4:**
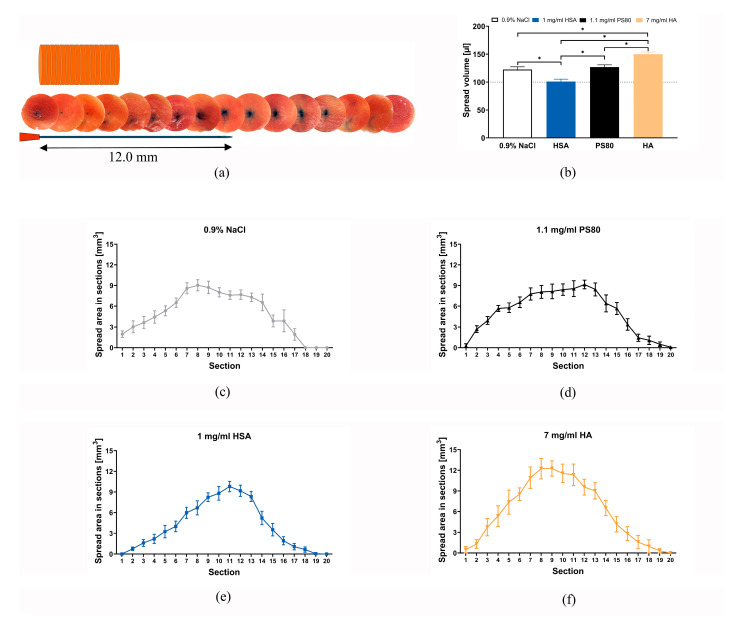
(**a**) Stack of cryosections taken from a porcine psoas major muscle column. Thickness of each section was 1.25 mm and diameter was 21.5 mm. In the displayed image stack 100 µL of 0.9% NaCl solution containing 0.025% bromophenol blue were injected through a 30-gauge cannula with 12 mm length using an electronic Eppendorf dispenser Multipette E3x with a 1 mL combitip at dispensing speed 3. The cannula was inserted centrally, as graphically indicated, parallel to the muscle fibers. After the injection the muscle column was frozen at −20 °C before it was sectioned. The injected solution spreads forward, backwards along the cannula, and sideways; (**b**) volumetric analysis of the spread volume detected in the muscle tissue after injection of stained formulations containing various polymers or 0.9% NaCl. Whereas the spread volume of the human serum albumin (HSA) formulation corresponded well with the injected volume of 100 µL, the spread volume of the NaCl 0.9% and the PS80 formulation (as in RELA) in the tissue was 122.6 µL and 127.5 µL, filling the existing interstitial space. The spread volume of the hyaluronic acid (HA) formulation of 150.0 µL was apparently larger because the viscous solution displaced the muscle fibers and created an additional liquid volume between the fibers, which was detected as stained volume (HA vs. saline, PS80 and HSA * *p* < 0.05 one-way ANOVA). Mean + SEM, *n* = 10. (**c**–**f**) Whereas the 0.9% NaCl and the PS80 formulations resulted in a 2D view as a plateau-shaped curve with an increase and decrease, the HSA and the HA formulations resulted in bell-shaped curves; in a 3D view, the spread of the 0.9% NaCl and the PS80 formulations appear more like a cylinder, and the HSA and the HA formulations like a ball. Mean ± SEM, *n* = 9–10.

Volumetric analysis of the spread volume visible in the muscle tissue after injection of stained formulations containing various polymers or 0.9% NaCl was used to compare the spread in muscle. Whereas the spread volume of the human serum albumin (HSA) formulation as in INCO corresponded well with the injected volume of 100 µL, the spread volume of the NaCl 0.9% and the PS80 formulation (as in RELA) in the tissue was larger (122.6 µL and 127.5 µL) but still filling the existing interstitial space. The spread volume of the non-commercial hyaluronic acid (HA) formulation (150.0 µL) was apparently larger because the viscous solution displaced the muscle fibers and created an additional liquid volume between the fibers which was detected by the image analysis as stained volume (Figure 4b).

Whereas the 0.9% NaCl and the PS80 formulations resulted in 2D view as a plateau curve with increase and decrease the HSA and the HA formulations resulted in bell-shaped curves; in 3D view the spread of the 0.9% NaCl and the PS80 formulations appear more like a cylinder and the HSA and the HA formulations like a ball (Figure 4c–f).

## 3. Discussion

An increasing number of BoNT/A products is approved and commercially available. For most indications, they are repeatedly injected at approximately 10- to 12-week intervals. More flexible and patient-tailored dosing regimens have been proposed for certain indications, which brings up the discussion on longer or shorter duration of effect of specific BoNT/A products [38].

In this study, we compared three modern 150 kDa BoNT/A products free of the BoNT/A complexing proteins INCO, DAXI and RELA in vitro. In addition, INCO and DAXI were compared in an in vivo paradigm because DAXI has been claimed by the manufacturer to last longer than previous neurotoxins [22,39]. The potential effects of different formulation or manufacturing processes on potency, duration of effect, specific activity or spread from the injection site were investigated.

INCO contains the least amount of BoNT/A protein per 100 units, RELA somewhat more (0.44 ng and 0.46 ng/100 U) and DAXI 32% more (0.58 ng/100 U), indicating that INCO comes with the highest specific activity, closely followed by RELA and with some distance DAXI (Table 2). The calculated specific potency was lowest in DAXI with 181.2 U/ng compared to INCO with 266.7 U/ng or RELA with 252.9 U/ng. However, due to the limited availability, only a single batch of DAXI could be analyzed in contrast to the three and four batches for RELA and INCO, respectively. The NT content determined for INCO is consistent with that of the 100 units vial (0.447 pg/vial) published previously [40]. In order to rule out effects on the ELISA detection arising from the different excipients in the formulations of DAXI and RELA, we tested the excipients, which turned out to not affect the ELISA validated for INCO.

Typical sandwich ELISAs like the one used here do not distinguish between active and inactive protein and recognize the amount of target protein irrespective of the correct three-dimensional structure. While monoclonal antibodies bind to a single epitope and polyclonal antibodies to several, neither reflects the biological activity of the protein. Thus, ELISA results can be misleading. Therefore, it is important to also determine the specific biological activity per 100 labeled units or per ml using an assay that reflects the entire molecular function, such as a cell-based potency assay [41,42] and finally calculate the specific activity of the protein.

Surprisingly, Lekholm et al. compared a 50 U RELA dose with a 20 U ONA dose for glabellar frown lines treatment [35] even though the toxins are considered to be similarly potent. The specific activities of INCO and RELA are similar and, e.g., ONA was shown to be as effective as INCO for therapeutic and aesthetic indications. Although units are in principle not interchangeable from a regulatory perspective, ONA and INCO are converted 1:1 in clinical practice [11,13,14,16,17,27]. For both products a dose-dependent prolongation of effect duration was demonstrated with doses above the recommended 20 U when treating upper facial lines [43,44]. Kerscher et al. reported in 2021 that increasing INCO from 20 U to 50 U prolonged the time-to-return to baseline in 75% of patients to 173 days vs. 119 days when treating glabellar frown lines [43]. A very similar duration of effect was observed with 50 U RELA for the treatment of glabellar frown lines in a clinical Phase 3 trial demonstrating that 75% of patients did not return to baseline 169 days after treatment [45]. These data underline that the clinical efficacy of RELA is not higher than INCO at equipotent dosing for the treatment of glabellar frown lines. This is consistent with the in vitro data in this study showing that the unit activities of INCO and RELA do not differ significantly. Accordingly, the RELA manufacturing technology does not yield a drug substance of superior potency when compared to INCO using similar conditions. The RELA excipient polysorbate 80 was concentration- and time-dependently cytotoxic for human lymphoma and keratinocytic cells [46], consistent with our findings in neuronal cells. It did not affect the neurons in our assay because polysorbate 80 or RTP004 were diluted 1:250 from the clinically provided concentrations; if added to the medium undiluted, they would have been detrimental to the neuronal cell membranes.

In vitro, DAXI showed a lower neurotoxin potency compared to INCO or RELA. It has been contemplated that due to the unique composition of excipients, especially the positively charged peptide RTP004, DAXI might have a higher potency and longer duration of effect in vivo [22,39]. In this study, INCO and DAXI were also compared in vivo for the local paralysis on the ipsilateral hind limb toe spread reflex of mice and for the spread visualized by in vivo imaging after injection into the right gastrocnemius muscle. IM administration of 20 U/kg INCO led to a more pronounced paralysis than DAXI at equivalent dose over the entire study period except for days 2 and 42. The paralytic effect of INCO was significantly higher from day 7–28 (* *p* < 0.05), indicating a higher potency of BoNT/A in this formulation. This stronger effect was not observed at the high BoNT/A dose 40 U/kg because the DAS reached its technical maximum (score 4) with both drugs, and the paralytical effect could not be further enhanced.

At 20 U/kg BoNT/A, the DAS response after INCO was not only more pronounced but also muscle paralysis lasted 10 days longer, which resulted in a 1.5-times higher DAS_AUC_ compared to DAXI. The increased local paralysis indicates a higher potency of INCO in this formulation, which may be due to a higher amount of active toxin molecules after reconstitution with saline or to a higher uptake of light chain at the neuromuscular junction. It does not support the hypothesis that the positively charged RTP004 peptide leads to an increased binding efficacy of the BoNT/A in DAXI to neuronal surfaces. Nor does it support a facilitated localization of the product for internalization and reduction in its diffusion from injection sites leading to increased uptake at the neuromuscular junction and prolonged efficacy as postulated in the literature. A reduced spread of DAXI would have led to reduced activity in the adjacent muscle in the DAS but reduced spread was not observed in the FRF imaging. In addition, a selective binding of DAXI to neuronal surfaces instead of other cells with negative surface charge due to exposed sialic acid residues on the surface glycans [47] was not demonstrated either.

BoNT/A-treated animals lost body weight, compared to placebo, dose-dependently and transiently until day 4 and day 7 post-injection, when administering 20 or 40 U/kg DAXI or INCO. This finding was also observed by other researchers after administering BoNT/A at this dose [48,49,50]. The reduced mobility due to the local muscle relaxation was suggested to hamper eating and drinking during the first days after the injection [37].

DAXI has come a long way; its active ingredient, the 150 kDa BoNT/A, is formulated with a synthetic peptide consisting of two protein transduction domains RKKRRQRRR corresponding to amino acids 49–57 of the HIV Tat protein (Swiss-Prot P04601.1) separated by a 15-amino acid polycationic linker. This HIV-1 Tat protein transduction domain (PTD) protein construct caused concentration-dependent cytotoxicity in human iPSC-derived neurons [51]. This is a cause for concern as the Tat protein has multiple effects advantageous for the HI virus but disadvantageous for the patient. Examples are the involvement in HIV-associated neurocognitive disorder by increasing NMDA receptor-mediated toxicity predominantly in the hippocampal CA1 region or accelerated aging [52]. The non-covalent mixture of BoNT/A and the polycationic peptide was first used in a clinical Phase 2 trial in 2008 and came in a gel formulation for topical administration for lateral canthal lines. Nine Phase 2 and 3 Phase 3 trials followed until 2015. The topical formulation was finally discontinued due to low effectiveness and, presumably, for safety concerns. Due to the low efficiency of transdermal transportation, thousands of units of BoNT/A would need to be applied to elicit an effect—laborious and risky compared to the parenteral standard therapy. The basic assumption behind this approach, that the Tat protein transduction domain included in topical formulation would efficiently transport the 150 kDa BoNT/A through the stratum corneum of the skin, was faulty. The formulation was then changed for lyophilization to allow for intramuscular injection and later named DaxibotulinumtoxinA-lanm; lanm stands for long-acting neuromodulator, which is an unproven claim of the manufacturer. PTD was now presumed to function as a polycationic bridge between the negatively charged botulinum toxin and the negatively charged extracellular matrix of presynaptic nerve terminals of the neuromuscular junction. This would assumedly inhibit the spread of neurotoxin from the injection site. Thus, avoiding local or systemic side effects with the option of higher dosing that would result in longer duration of effect. So, importantly, DAXI has always been considered as equipotent with ONA [22,39]. In this study, for in vivo imaging, the DAXI formulation was reiterated with an inactive BoNT/A mutation [53] labeled with the fluorescent dye Alexafluor-647. Although the polycationic peptide was added at 7.7 times higher concentration than in reconstituted DAXI, the spread from the injection site was indistinguishable from the HSA formulation used in INCO. In order to demonstrate the effect of a confining formulation that effectively reduces the neurotoxin spread from the injection site, we compared the injection of the same amount of neurotoxin in a non-commercial hyaluronic acid-containing formulation. This resulted clearly in a slower and smaller spread indicating that an appropriate formulation could materialize the claims. In clinical trials on glabellar lines 40 U DAXI were compared to 20 U ONA which resulted in ~25% longer duration of effect (≥1-point improvement on Investigator Global Assessment—Facial Wrinkle Scale) [26,27,54]; a comparison with ONA 40 U was not conducted but would have been more appropriate because neither 40 U ONA nor 50 U INCO as a 150 kDA comparator BoNT/A would have caused serious treatment-emergent adverse events and also have lasted longer [43,55,56]. DAXI for plantar fasciitis, a pain indication, was discontinued presumably due to low effectiveness and a strong placebo effect [57,58], scientific publications are not available.

In a clinical Phase 3 study on cervical dystonia a consistent dose–response relationship could not be demonstrated for DAXI; again, a plausible explanation has not been provided. The time to loss of ≥80% peak treatment benefit was 20.3 weeks when treating patients with 240 U DAXI and 24 weeks when treating patients with 125 U [28].

In vivo imaging using fluorescence-labeled INCO or DAXI did not show any difference in area during the observation time of 48 h after injection. Thus, the claim that DAXI would spread less [22] in muscle tissue due to the polycationic peptide excipient could not be confirmed. However, confinement of BoNT/A at the injection is achievable by, e.g., a hyaluronic acid-containing formulation which stayed for about double the time at the injection site compared to INCO or DAXI. The spread of RELA which was studied ex vivo in porcine muscle tissue was compared to INCO or NaCl 0.9%. Spread is not a clearly defined term; it describes the physical distribution in the tissue interstitial volume depending on injection volume, pressure, speed and viscosity. It was larger than the INCO spread and equal to the NaCl 0.9% spread, indicating that the polymer HSA keeps the injected liquid volume more confined than just a NaCl 0.9% solution. Interestingly, RELA, which is formulated with polysorbate 80, also a polymer, spread like NaCl 0.9%. The reason could be a too low amount of PS80 to achieve such a confining effect. A higher concentration of PS80 than 1.1 mg/mL—as in RELA—would, however, be even more cytotoxic on neuronal cells and thus not be advisable [51]. The burning injection pain reported by 7.2% of the patients injected with RELA [59] may reflect the local transient cytotoxicity of PS80.

Together with our preclinical analytical and in vivo data it is concluded that DAXI is at best equipotent with INCO, ONA, or RELA. A longer duration of action resulted, if demonstrated by other authors on glabellar frown lines and CD, from a higher dose. There is no convincing evidence for an improved benefit–risk ratio as compared to other BoNT/A formulations such as INCO, ONA, or RELA, although the idea of combining BoNT/A with a polycationic Tat-PTD peptide sounds understandable; however, DAXI does not materialize on it.

A limitation of our study is that only one batch of DAXI was available for the investigations. More batches were unavailable for us at the time of material acquisition. The present study investigated the pharmacological effects using a single injection of DAXI and INCO only. Repeated dosing at various intervals would allow a more comprehensive potency comparison.

In summary, we could demonstrate in vitro by ELISA and CBA analysis that the unit activity for INCO and DAXI is comparable. The calculated specific neurotoxin activity of DAXI was lower. The analytical data on RELA support the clinical data showing a comparable efficacy of RELA and INCO [43,44,45] and does not support the hypothesis that the manufacturing technology would lead to a superior potency of RELA compared to INCO or ONA. We showed that the in vivo efficacy of INCO is higher compared to DAXI if used at doses below technical ceiling, which supports the assumption that RTP004 does not lead to more cell surface binding of the neurotoxin at nerve terminals. Also, RTP004 did not keep the injected BoNT/A confined to the injection site. In the end, the BoNT/A effect depends on the administered bioactivity, measured in dose units. The excipients polysorbate 80 or RTP004 in new BoNT/A products do not provide an advantage but give cause for concern due to the cytotoxicity reported in the literature.

## 4. Conclusions

While similar levels of biological activity were found in the three products, INCO and RELA contain less BoNT/A than DAXI. This indicates that DAXI contains a substantial amount of inactive BoNT/A compared to the other two products.

In the in vivo comparison of INCO and DAXI, INCO elicited a significantly higher paralytic effect with a prolonged duration of action after application of 20 U/kg. These findings suggest that INCO may offer better overall therapeutic performance than DAXI in clinical settings.

## 5. Materials and Methods

### 5.1. Botulinum Toxin A Formulations

The complexing protein-free 150 kDa BoNT/A products INCO (batch No. C00024920, C00023640, C00025190, C00024770), DAXI (batch no. R1005125; only one batch available upon analysis) and RELA (batch no. 23093-1, 23256-1, 23310-3) were studied. Lyophilizates in 100 U/vials (INCO or DAXI) were reconstituted in saline without preservatives (0.9%, B. Braun AG, Melsungen, Darmstadt, Germany) to obtain 20 or 40 U/mL for the DAS assay. The control group was treated with the same volume of saline. The detailed composition of each BoNT/A formulation is listed in Table 1.

For in vivo imaging, an inactive E224A/E262A mutant full-length BoNT/A [53] was labeled with the far-red fluorescent dye Alexa Fluor 647 (Thermo Fisher Scientific Inc., Le Genest-Saint-Isle, French and Sulzfeld, German/Life Technologies, Darmstadt, Germany, #A20006) according to the manufacturer instructions. The labeled protein (fiBoNT/A) was diluted to a final concentration of 50 µg/mL in the respective excipient matrix of INCO or DAXI (see Table 1, except 75 µg/mL RTP004 for DAXI) or in a formulation containing 0.9% NaCl and 5 mg/mL 3.3 MDa sodium hyaluronate (non-commercial HA formulation).

### 5.2. Animals

For digital abduction scoring male Balb/c mice (adult, 5–7 weeks old; Janvier Labs, Le Genest-Saint-Isle, France) were used. For in vivo imaging female SKH1 hairless mice (5–7 weeks; Charles River Labs, Sulzfeld, Germany) were used. Animals were kept in an enriched environment, at 22 ± 2 °C temperature, 30–70% humidity, 12 h light–dark cycle, food and water ad libitum. Animals had at least 7 days to settle before the beginning of the study. Animal procedures were compliant with the European Communities Council Directive 2010/63/EU. Study protocols were approved by the Local Committee for animal studies of Brandenburg (2243/2-13-2024-19-G or 2347-6-2018).

### 5.3. Experimental Protocol for DAS

Prior to BoNT/A administration the animals were anesthetized with 5% isoflurane gas in 100% medical oxygen.

The BoNT/A products or placebo (saline 0.9%) were injected once into the right gastrocnemius muscle at a BoNT/A dose of 20 or 40 U/kg in 20 µL of volume. Reliable potency indicating parameters in the DAS are the peak score and the duration of paralysis at a certain effect level. In total, 0.8 U/20 g mouse is the dose in our paradigm, which causes systemic toxicity but no lethality and, at the same time, a maximum local paralysis score of 3.5–4. Lower doses and scores can be done but complicate the comparison of effects. The volume was tested in mice of this strain and bodyweight and did just not leak out of the muscle. It was based on the experience of other groups who injected 25 µL in the mouse tibialis anterior muscle [60]. For the IM injection the right hindlimb was shaved using an electric razor (Aesculap AG, Tuttlingen, Germany) and the injection was done with a 100 µL Hamilton syringe (Hamilton Company, Reno, NV, USA) with a 30-gauge needle (B. Braun AG, Melsungen, Germany) into the lateral gastrocnemius head but flooding the entire muscle. The injection was apparently not painful. After injection, the mice were warmed up with an infrared lamp and returned to their cage. The physical condition of the animals was checked daily throughout the experiment. The experiment was terminated by sacrificing the animals by cervical dislocation in deep anesthesia.

Body weight was measured every 1–4 days throughout the study. On day 38 one moribund animal in the INCO 20 U/kg group was euthanized for humane reasons; in the other groups all animals completed the study.

#### DAS Assay and Body Weight Development in Mice

The hind limb digit abduction reflex was induced by lifting up the mice at the back. No mouse was excluded for an irregular DAS response before beginning of the study. For the scoring, a five-point scale from normal (DAS 0) to full inhibition (DAS 4) was used as described before [36]. The score was read in 0.25 steps as established by the author [61]. The score was read at 1–4 days intervals over 25 days after the BoNT/A injection, always by the same experimenter. The relative body weight was calculated against the bodyweight measured before the BoNT/A injection as the denominator.

### 5.4. Determination of Concentration and Biologic Activity of BoNT/A Products

The neurotoxin content in both products was studied by a Sandwich-ELISA as follows: Microtiter plates (Nunc, Roskilde, Denmark) were coated with an equine anti-BoNT/A antibody (NIBSC Botulinum Antitoxin Type A 59/021, Hertfordshire, UK) which captures and immobilizes the BoNT/A contained within the samples applied to the plates. Subsequently, BoNT/A binds a rabbit anti-BoNT/A detection antibody (Merz Therapeutics, Frankfurt, Germany) and the detection antibody is bound by a horse-reddish peroxidase-coupled donkey anti-rabbit IgG secondary antibody (Thermo Fisher Scientific #31458, Darmstadt, Germany). After washing, a substrate is added (1-Step Ultra TMB-ELISA Substrate Solution, Thermo Fisher Scientific # 34028, Darmstadt, Germany) and the enzyme generates a colored reaction product proportional to the amount of BoNT/A contained in the samples allowing the quantification by comparison to a calibration curve.

The ELISA used for BoNT/A concentration measurements is validated for measurement of INCO. As DAXI and RELA contain excipients with potentially unknown effects in the ELISA, spiked samples of the respective formulations were tested for equivalence in order to ensure validity of the reported results. For each of the products, the same amount of BoNT/A was added to solutions with the same excipient matrix as contained in the samples prepared for measuring BoNT/A concentration in DAXI, RELA or, the validated sample matrix of the ELISA. BoNT/A spiking to the solution was controlled by weighing and the resulting concentration measurement corrected for the weighing result. At least 11 valid measurements were obtained for each formulation. Equivalence testing was performed using the software OriginPro (Origin^®^ 2019b, OriginLab Corporation, Northampton, MA, USA). Briefly, for each of the two test products, the difference in measurement results of test matrix vs. validated matrix was calculated. At a significance level of 0.05, the measured BoNT/A concentrations of the samples composed as the DAXI or RELA samples were equivalent within the limits of +/−7% of the concentrations determined in the samples with validated sample matrix.

For potency measurements of INCO, DAXI and RELA, a modified in vitro potency assay based on neuronal cells (Fujifilm Cellular Dynamics, Inc., Madison, WI, USA) was used [41]. After thawing, the cells were seeded on 96-well plates (Corning, NY, USA) and incubated for 24 h in 5% CO_2_ humidified air at 37 °C. The following day, medium (Fujifilm Cellular Dynamics, Inc., Madison, WI, USA) was changed, and the cells were cultivated for 72 h. The cell culture medium was replaced by medium containing different concentrations of BoNT/A and then cultivated for 72 h. During this time the toxin can cleave intracellular SNAP25. Subsequently, the cells were fixed and permeabilized (20 min) in the microtiter plate (Corning, NY, USA). Using ELISA, the amount of SNAP25 cleaved by BoNT/A, as well as the amount of total SNAP25 was determined and the ratio was calculated. Both targets were detected by the anti-BoNT/A cleaved SNAP-25 monoclonal antibody produced in mouse (Merz Pharmaceuticals GmbH, Frankfurt, Germany) and anti-SNAP-25 polyclonal antibody produced in rabbit (Sigma, Taufkirchen, Germany) in one well and quantified by secondary antibodies HRP-conjugated polyclonal goat anti-mouse IgG and alkaline phosphatase-conjugated polyclonal donkey anti-rabbit IgG (Jackson ImmunoResearch, distributed by Dianova, Hamburg, Germany). The ratio of total protein to cleavage product is a measure of the biological BoNT/A activity. To enhance the sensitivity to BoNT/A, cells were treated with trisialoganglioside GT1b (Cayman Chemical distributed by Biomol, Hamburg, Germany) three days before and during exposure to BoNT/A. By comparing reference standard versus sample a relative potency ratio was calculated through parallel line analysis (PLA3.0, Stegmann Systems, Rodgau, Germany).

### 5.5. In Vivo Imaging in Mice

In total, 20 µL of either formulation of labeled, inactive BoNT/A were injected into the right gastrocnemius muscle of female SKH-1 hairless mice. For far-red fluorescence imaging (FRF imaging), the mice were anesthetized with 2% isoflurane and placed face down on the table of the image station with continued anesthesia. Images were taken prior to injection of fiBoNT/A and at distinct time points over a period of 48 h post-injection at 627 nm excitation light and 660 nm emission filter using an Andor iKon-M camera (Andor Technology Ltd., Belfast, UK). Corresponding to each fluorescence picture, a visible light photograph was taken to document the exact positioning of the animal.

The resulting images were analyzed using Andor Solis V. 4.27 software (Andor Technology Ltd., Belfast, UK). For quantitation of the fluorescence signal, an area of 70 × 106 pixels at the injection site was selected with the brightest fluorescence of the image in the center. The brightness values of these 7041 pixels were summarized yielding the total fluorescence intensity in this area. The fluorescence at t = 0 (immediately after injection) for each sample was set to 100% and the data of the following time points were normalized to this value. For visualization of the results, the fluorescence intensity pixel values were pseudo-color converted and overlayed with the position control photograph.

### 5.6. Ex Vivo Investigation of BoNT/A Spread

In order to compare the volume of BoNT/A spread after INCO or RELA IM injection the spread after injection into muscle tissue was studied in an ex vivo experiment. To create consistent muscle tissue specimens, cylinders of 21.5 mm diameter and 30 mm length in muscle fiber direction were dissected from porcine psoas major muscle. Then, 100 µL of each polymer formulation (human serum albumin for INCO and PS 80 for RELA) or 0.9% NaCl solution containing 0.025% bromophenol blue was injected IM into a muscle cylinder using a 1 mL syringe with a 30-gauge needle with 12 mm in length (Figure 4a). For constant and reproducible injection speed, an electronic Eppendorf dispenser Multipette E3x with a 1 mL combitip (Eppendorf, Germany) set to dispensing speed 3 was used. The cannula was placed in parallel to the muscle fibers into the tissue with its full length. Ten minutes after the injection, the muscle cylinders were frozen at −20 °C and after 1 h cut into 1.25 mm thick transversal sections. Toxin spread was analyzed by measuring the stained distribution area in each cryosection using ImageJ 1.54p, and the distribution volume was calculated by stacking the sections.

### 5.7. Life–Dead Assay

To prove the compatibility of polysorbate 80 and RTP004 with the neuronal cell cultures used for the potency assay in this study, they were exposed to these excipients at various concentrations and time periods and subsequently subject to a Life–Dead Assay. iCell neurons (Fujifilm Cellular Dynamics, Inc., Madison, WI, USA) were seeded at a density of 300,000 cells/mL in 96-well plates (Corning, NY, USA) and incubated for 24 h at 37 °C in a humidified atmosphere containing 5% CO_2_. After this initial incubation, the culture medium was replaced, and cells were maintained under the same conditions for an additional 72 h. Subsequently, cells were treated with various test compounds for three different incubation durations: 72 h, 4 h, and 1 h. The tested substances whose concentrations upon IM injection are as follows: polysorbate 20 (0.083 mg/mL) (Sigma-Aldrich, Steinheim, Germany), polysorbate 80 (0.733 mg/mL) (Merck KGaA, Darmstadt, Germany), D-(+)-trehalose dihydrate (30 mg/mL) (Sigma-Aldrich, Steinheim, Germany), L-tryptophan Biochemica (0.667 mg/mL) (AppliChem GmbH, Darmstadt, Germany), L-histidine, U.S.P. (0.555 mg/mL) (Avantor Performance Materials, LLC, Matsonford, PA, USA), and sodium dihydrogen phosphate dihydrate (1.514 mg/mL) (Merck KGaA, Darmstadt, Germany). Starting from these concentrations each compound was tested at the dilution level of 1:250 as used in the in vitro cell-based potency assay. In addition, the combinations of these compounds as contained in the commercial formulations of DAXI and RELA were investigated at the same dilution level. Cell cytotoxicity and viability was subsequently assessed using the CellTox™ Green Cytotoxicity Assay (Promega, Madison, WI, USA) and the CellTiter-Glo^®^ 2.0 Assay (Promega, Madison, WI, USA). Fluorescence and luminescence signals were measured using a microplate reader (Synergy H1, Agilent BioTek, Santa Clara, CA, USA). All procedures were performed in accordance with the respective technical manuals provided by the manufacturer.

### 5.8. Statistics

The group mean body weight was expressed as percentage of the initial body weight before injection. Data were analyzed by two-way repeated-measures analysis of variance (RM ANOVA) followed by Dunnett’s multiple comparisons analysis as post hoc test. RM ANOVA was applied to assess the potency of BoNT/A in the DAS over time. Comparisons between groups were performed using Dunnett’s multiple comparisons test when significant main effects were observed in the RM ANOVA. If values were missing, the mixed-effects model was applied, followed by Šídák’s multiple comparisons test.

An unpaired, one-tailed non-parametric Mann–Whitney test was applied to compare two groups. The mean area under the curve was calculated (DAS_AUC_) for DAS over time curves. Two groups were compared by the unpaired, parametric *t*-test with Welch’s correction.

Data are presented as mean ± SEM. Descriptive and interference statistics were calculated with GraphPad Prism (version 9.5.1, GraphPad Software, San Diego, CA, USA). The level of significance was α = 0.05.

## Figures and Tables

**Table 1:** Differences in excipients, concentration, and biologic activity in the three BoNT/A products analyzed in this study. Data are shown as means +/− SD where applicable.

Product	IncobotulinumtoxinA	DaxibotulinumtoxinA	RelabotulinumtoxinA
Dosage (U) per vial	100	100	150
API	Purified toxin (150 kDa)	Purified toxin (150 kDa)	Purified toxin (150 kDa)
Volume	0.5–5 mL (Xeomin US PI and EU SPC) 2–2.5 mL (Bocouture US PI)	1.2 mL (Daxxify US PI)	1.5 mL (Relfydess AUS PI)
Appearance	Lyophilizate	Lyophilizate	Liquid
Formulation	HSA 1.0 mg Sucrose 4.7 mg	L-Histidine 0.14 mg L-Histidine-HCl monohydrate 0.65 mg Trehalose dihydrate 36 mg Polysorbate 20 0.1 mg RTP004 peptide 11.7 µg	NaH_2_PO_4_ dihydrate 0.9 mg/mL Na_2_HPO_4_ dihydrate 0.7 mg/mL KCl 0.2 mg/mL NaCl 8.2 mg/mL Polysorbate 80 1.1 mg/mL Tryptophan 1.0 mg/mL H_2_O for injection
Total neurotoxin protein (ng per 100 U *)	0.44 ± 0.04	0.58	0.46 ± 0.01
Relative biological activity (U/vial)	116.00 ± 8.16	104.21	116.79 ± 5.45
Specific neurotoxin potency (U/ng)	266.68 ± 19.97	181.12	252.89 ± 7.62

* Units of measurement for the three commercially available BoNT/A preparations are proprietary to each manufacturer and are not interchangeable. Abbreviations: API, active pharmaceutical ingredient; PI, prescribing information; SPC, summary of product characteristics; HCl, hydrogen chloride; HSA, human serum albumin.

**Table 2:** Digit abduction score (DAS) response observed on the injected leg after intramuscular administration of different BoNT/A doses.

Treatment	Dose U/kg	Mean Peak DAS	Duration of Action (d)	DAS_AUC_ (DAS * d)
INCO	20	2.88 ± 0.15	21.0	29.00 ± 3.43 *
DAXI	20	2.97 ± 0.13	11.0	19.13 ± 2.16
INCO	40	3.78 ± 0.13	26.4	51.25 ± 5.64
DAXI	40	3.94 ± 0.06	24.7	53.25 ± 1.94

Data expressed as mean ± SEM. Unpaired *t*-test * *p* < 0.05; DAS_AUC_: Total DAS response over time. Duration of action: Mean time-to-return to DAS = 0.5.

## Data Availability

The original contributions presented in this study are included in the article. Further inquiries can be directed to the corresponding author(s).

## Abbreviations

The following abbreviations are used in this manuscript:

ABO	AbobotulinumtoxinA
BoNT/A	Botulinum toxin type A
CBA	Cell-based assay
DAS	Digit abduction score
DAS_AUC_	Total DAS response versus time
DAXI	DaxibotulinumtoxinA
HIV-1	Human immunodeficiency virus
IM	Intramuscular
INCO	IncobotulinumtoxinA
ONA	OnabotulinumtoxinA
PTD	Protein transduction domain
RELA	RelabotulinumtoxinA
